# A Hybrid TGfU/SE Volleyball Teaching Unit for Enhancing Motivation in Physical Education: A Mixed-Method Approach

**DOI:** 10.3390/ijerph18010110

**Published:** 2020-12-26

**Authors:** Alexander Gil-Arias, Sergio Diloy-Peña, Javier Sevil-Serrano, Luis García-González, Ángel Abós

**Affiliations:** 1 Centre for Sport Studies, Rey Juan Carlos University, Fuenlabrada, 28943 Madrid, Spain; 2 EFYPAF “Physical Education and Physical Activity Promotion” Research Group, Faculty of Health and Sport Sciences, University of Zaragoza, 22002 Huesca, Spain; sdiloy@unizar.es (S.D.-P.); jsevils@unizar.es (J.S.-S.); lgarciag@unizar.es (L.G.-G.); 3 EFYPAF “Physical Education and Physical Activity Promotion” Research Group, Faculty of Social Sciences and Humanities, University of Zaragoza, 44003 Teruel, Spain; aabosc@unizar.es

**Keywords:** pedagogical models, hybridization, models-based practice, gender, self-determination theory

## Abstract

Grounded in self-determination theory, this pre-experimental study analyzed the effects of a hybrid teaching games for understanding/sport education (TGfU/SE) volleyball teaching unit on students’ motivational outcomes, using a mixed-method approach. It also examined whether the intervention was equally effective for boys and girls. Participants were 53 secondary school students (*M_age_* = 15.50, *SD_age_* = 0.57) who were taught through a hybrid TGfU/SE unit. The structure of this unit was designed according to the characteristics of SE model, while learning tasks were designed by using the pedagogical principles of TGfU model. Both self-reported validated questionnaires and focus groups were used before and after intervention to assess students’ motivational responses. After the hybrid TGfU/SE unit, both quantitative and qualitative findings showed improvements in students’ perceptions of need-support from the physical education (PE) teacher, basic psychological needs satisfaction, novelty, and variety satisfaction, as well as intrinsic motivation compared to baseline values. Although the hybrid TGfU/SE unit was effective in both genders, a large effect size was found for girls. Despite the existence of social and cultural stereotypes in team sports such as volleyball in favor of boys, results highlight the importance of developing hybrid TGfU/SE units to improve students’ motivational outcomes, especially in girls.

## 1. Introduction

In the last decade, models-based practice (MBP) has become a leading trend for teachers and researchers alike in physical education (PE) contexts [1]. Recently, MBP has been identified as an alternative pedagogical approach to a teacher-centered direct instruction model, which permits moving PE beyond an activity-driven view of curriculum to a models-based instructional approach to teaching and learning [2]. Consequently, MBP offers students broader and more in-depth teaching through student-centered approaches, which address achieving various learning outcomes (e.g., physical, cognitive, social, and affective) beyond learning, through one single pedagogical model [3,4].

Despite this, research tends to focus on the delivery of a single model, especially teaching games for understanding (TGfU) [5] or sport education (SE) [6]. Such has been this prevalence of single model research and practice that MBP has become synonymous with single model [2]. For this reason, the common characteristics shared by several pedagogical models, the need to overcome limitations in educational contexts, and the belief that no single model can be adapted to all content areas, have led PE teachers to combine different pedagogical models or parts of them [4]. Certainly, both TGfU and SE models provide a learning environment where the use of purposeful, authentic, and meaningful tasks can easily be combined to provide developmentally appropriate games experiences for students [7].

Within the TGfU model, students learn to play the game prioritizing the understanding of tactics and strategies. To reach an optimal understanding of the game, the TGfU model is based on representative (e.g., simplified and/or small-sided) and exaggerated (e.g., modified/conditioned) versions of the game [8]. As the TGfU model prioritizes the cognitive learning domain using the “what” before the “how”, there may be concerns that technical skills are either ignored or left underdeveloped [9]. Yet, in TGfU lessons, students’ technical skills are developed in two ways: (a) alongside tactics in the contextualized situations of small-sided and/or modified/conditioned games; and/or (b) they are practiced when needed within “skill drills” that are planned and set up in lessons by the teacher [10].

The SE model aims to offer students a meaningful sport experience; one that is close to regulated sports competitions but maintains the educational values inherent to PE. The SE model was developed due to the desire to create authentic and educational practices for all PE students, aiming to produce competent, literate, and enthusiastic students [11]. To get a successful implementation, PE teachers must follow six key features [12]: (1) seasons, (2) persistent teams, (3) formal competition (in developmentally appropriate small-sided and/or modified/conditioned games), (4) roles other than player (e.g., coach, captain, statistician, etc.), (5) record-keeping, and (6) festivity. The implementation of these six characteristics makes the SE model a highly inclusive experience for all types of students [12]. In this sense, all participants have sufficient opportunities to experience social interactions [13,14], especially girls and less skillful students, who also feel that they can make valuable contributions to their teams [15].

Although TGfU and SE have different features, both pedagogical models share several common objectives, concepts, and pedagogical processes. For example, both pedagogical models are consistent with contemporary instructional approaches, which include an outcome-based education, cooperative learning, and peer tutoring. Moreover, learning within these pedagogical models is closely aligned with the constructivist theories of learning, and therefore, students are perceived as active, social, and creative learners who build their own knowledge, and identify what they need to improve during the learning process [7]. Likewise, although each model has its own limitations when applied independently, the hybridization of these models can help PE teachers to achieve higher quality learning outcomes [2,3]. Based on this, hybridization could be considered an innovative trend in PE. This new pedagogical approach may provide researchers and practitioners with a deeper understanding of how students’ perceptions of the learning environment created by PE teachers influence their response patterns (e.g., engagement and motivation) when teachers use an MBP [4].

Within the educational context, self-determination theory (SDT) has been established as one of the most reliable theoretical frameworks through which to explore and understand students’ motivational processes [16,17]. According to SDT, the learning environment created by PE teachers may influence the satisfaction of three innate and universal human needs of autonomy (e.g., desire to commit to an activity due to one’s own choice), competence (e.g., desire to interact efficiently with the medium to feel competent), and relatedness (e.g., desire to feel part of the group) [16,17,18]. More precisely, PE teachers can satisfy the students’ basic psychological needs (BPNs) via a need-supportive teaching style, characterized by autonomy support (e.g., gradually passing on decision-making and responsibility to students), competence support (e.g., adapting activities to the level of the students’ ability), and relatedness support (e.g., facilitating group and collaborative activities) [19,20]. The satisfaction of the three BPNs may determine the type of motivation that students have in their PE classes [16,17,18,19,20,21]. SDT proposes that motivation lies along a continuum, from more to less self-determined forms of motivation. Intrinsic, integrated, and identified regulations represent the more autonomous forms of motivation (e.g., engaging in an activity due to internal reasons such as personal values, benefits, life goals, or pleasure), while introjected and external regulations represent the more controlled forms of motivation (e.g., participation is focused on achieving other objectives such as social recognition, praise, and/or external rewards). The continuum concludes with amotivation, which is the lowest level of self-determination and refers to the absence of intention or reasons for participating in PE [16].

Previous studies in a PE context have indicated that students’ BPN satisfaction is related to autonomous motivation [20]. In recent years, research has begun to examine other variables which could benefit autonomous motivation of students, such as novelty (e.g., students’ sense of experiencing something new or unusual), and variety (e.g., students’ sense of experiencing a combination of novel and familiar tasks) [18,22,23,24]. Finally, autonomous motivation, contrary to controlled motivation and amotivation, is related to students’ positive affective (e.g., enjoyment), cognitive (e.g., academic achievement), or behavioral (e.g., intention to be physically active) [25,26] outcomes. Consequently, in the PE context, it seems essential to investigate how MBP (models-based practice) (e.g., hybrid TGfU/SE unit) may affect students’ motivation. This becomes even more relevant given that PE teachers have traditionally expressed difficulties motivating their students to participating in PE [27].

Only a few previous existing studies have examined the effects of a hybrid TGfU/SE unit on student outcomes in PE lessons. A first study conducted by Hastie and Curtner-Smith [28] found that the act of teaching through a hybrid TGfU/SE model was labor-intensive and required the PE teacher to possess high levels of pedagogical content knowledge. Moreover, Portuguese researchers reported significant improvements in behavioral outcomes (e.g., decision-making and skill execution) when students were taught through a hybrid unit of SE/invasion games competence model (IGCM) or SE/step game approach (SGA; both of which share a similar conceptual structure to TGfU) [29,30].

Despite this increase in hybrid TGfU/SE model studies in recent years, some gaps still remain. First, the amount of research conducted to assess the impact of a hybrid TGfU/SE model on motivational outcomes is laughable compared to other kinds of outcomes [4]. This is surprising given that previous research findings reported that pedagogical approaches incorporating features of TGfU or SE are superior to the traditional approach in the facilitation of students’ BPN satisfaction and intrinsically motivated behaviors [13,31,32,33]. However, it is also true that some of the results of TGfU or SE model in terms of needs-support and autonomy and competence satisfaction are not totally conclusive [13], so further research on motivational variables seems required. Likewise, to the best of our knowledge, there are only three previous studies where researchers reported significant improvements in motivational variables related to SDT when Spanish secondary [34,35] and elementary [36] school students participated in a hybrid TGfU/SE unit. In addition, research that has examined the effects of the TGfU or SE model on novelty satisfaction is still scarce [37], and there is no study that has examined the effects of TGfU or SE model on variety satisfaction.

Second, to the authors’ knowledge, research that has examined whether the effectiveness of TGfU and/or SE models may differ depending on the students’ gender is practically lacking [13,14,15,16,17,18,19,20,21,22,23,24,25,26,27,28,29,30,31,32,33,34,35,36]. Although SDT postulates that male and female students have the same psychological needs, previous research in PE has demonstrated that gender can significantly influence students’ engagement in, and attitude towards, PE [38], with some girls feeling that they have fewer opportunities to play than their male counterparts [39,40]. In addition, researchers have argued that the PE context is stereotyped, since boys generally like sports and physical activities to entail speed, face-to-face competition, and bodily contact, while girls prefer non-aggressive sports [41]. In this regard, the impact of these gender stereotypes on students’ motivational beliefs should be considered within the design of experimental studies [42]. Finally, TGfU/SE studies conducted so far have been carried out from a quantitative or qualitative approach, which, in some cases, limits the understanding of the findings. From a critical point of view, and with the aim of adding to past TGfU/SE evidence, the research studies conducted should rely on a mixed-method approach to obtain more in-depth insights into students’ motivational processes, beyond those that can be gathered through quantitative or qualitative data alone [43].

Consequently, the present study aims to fill these three gaps in the existing research literature. Grounded in SDT and relying on a mixed-method design, the primary aim of this study is to examine the effect of a hybrid TGfU/SE volleyball teaching unit on a series of motivational outcomes. Additionally, this study also aims to assess the extent to which this hybrid TGfU/SE volleyball teaching unit is equally effective for both boys and girls. In this regard, and despite social and cultural stereotypes in team sports in favor of boys [41], it was hypothesized that teaching volleyball in PE through a hybrid TGfU/SE unit allows to design a more equitable and inclusive learning environments, where boys and girls would report higher post-test scores on motivational-related variables compared to baseline values.

## 2. Materials and Methods

### 2.1. Design and Participants

Within a mixed-method approach, this pre-experimental study relied upon a quantitative and qualitative research perspective with methodological triangulation [43]. Through purposive sampling, 53 students in their fourth year at secondary school (*M_age_* = 15.50, *SD_age_* = 0.57; *n* = 16 female), and from two different school groups attending one north-eastern Spanish public secondary school, were taught through a hybrid TGfU/SE volleyball teaching unit. The sample selection was determined considering the curricular plan of the school and the availability of the PE teacher to conduct a hybrid SE/TGfU volleyball teaching unit. For this study, the selection criteria of the sample were the following: (1) attending 8 of the 10 lessons that comprised the intervention; and (2) responding to all the questionnaires related to the study variables before and after the intervention. Importantly, students in both groups had no previous experience with TGfU and SE models, or in volleyball in PE lessons. Nevertheless, all participants had previous PE experience in other team sports (e.g., handball, basketball, football, etc.), which they were taught through direct instruction.

### 2.2. Measures

Data were recruited both through a set of validated questionnaires (e.g., quantitative methodology) and through focus groups (e.g., qualitative methodology) before and immediately after the hybrid TGfU/SE volleyball teaching unit. Because students had no previous experience in a volleyball PE teaching unit, the first data collection (pre-test) referred to their experiences in previous PE teaching team sport units, while the second data (post-test) collection referred to the volleyball teaching unit.

#### 2.2.1. Quantitative Measurement

Questionnaires were administered in paper-and-pencil format in the absence of the PE teacher who implemented the intervention. The time to complete the questionnaires was approximately 30 min.

##### Need-Supportive Behaviors from PE Teacher

Students’ perceptions of autonomy, competence, and relatedness support from the PE teacher were assessed using the Spanish version of the questionnaire of BPNs support in PE [44]. The scale begins with the question stem, “In team sport (pre-test)/volleyball (post-test) PE lessons, my PE teacher…” and includes 12 items (four items per factor) that measure autonomy support (e.g., “often asks us about our preferences with respect to the activities we carry out”; αpre/post = 0.60/0.70), competence support (e.g., “offers us activities based on our skill level” αpre/post = 0.76/0.75), and relatedness support (e.g., “encourages positive interactions among all pupils” αpre/post = 0.65/0.61). Due to the reduced number of items for each factor, Cronbach’s alpha values above 0.60 can also be accepted [45]. Responses were registered on a 5-point Likert scale ranging from 1 (“strongly disagree”) to 5 (“strongly agree”).

##### Basic Psychological Need Satisfaction

Students’ perceptions of autonomy, competence, and relatedness satisfaction were assessed using the Spanish version of the BPNs in exercise scale in PE [46]. The scale begins with the question stem, “In the team sport (pre-test)/volleyball (post-test) PE lessons…” and includes 12 items (four items per factor) that measure autonomy satisfaction (e.g., “I have the opportunity to choose how to perform the exercises”; αpre/post = 0.61/0.60), competence satisfaction (e.g., “I carry out the exercises effectively”; αpre/post = 0.81/0.80), and relatedness satisfaction (e.g., “I feel very comfortable when I do exercise with other colleagues”; αpre/post = 0.70/0.74). Due to the reduced number of items for each factor, Cronbach’s alpha values above 0.60 can also be accepted [45]. Responses were registered on a 5-point Likert scale ranging from 1 (“strongly disagree”) to 5 (“strongly agree”).

##### Novelty Satisfaction

Students’ perceptions of novelty satisfaction in PE were assessed using the Spanish version in PE of the novelty need satisfaction scale [24]. This one-factor questionnaire comprises five items (e.g., “I frequently feel there are novelties for me”; αpre/post = 0.80/0.82) preceded by the stem: “In the team sports (pre-test)/volleyball (post-test) PE lessons...” Responses were registered on a 5-point Likert scale ranging from 1 (“strongly disagree”) to 5 (“strongly agree”).

##### Variety Satisfaction

Students’ perceptions of variety satisfaction in PE were assessed with an adapted version of the perceived variety in exercise scale [47]. This one-factor questionnaire comprises five items (e.g., “I feel like my PE lessons are varied”; αpre/post = 0.74/0.75) preceded by the stem: In the team sports (pre-test)/volleyball (post-test) PE lessons...” Responses were registered on a 6-point Likert scale ranging from 1 (“strongly disagree”) to 6 (“strongly agree”).

##### Motivation

Students’ perceptions of different motivational regulations in PE were assessed using the Spanish version in PE of the perceived locus of causality questionnaire [48]. The scale begins with the question stem, “I engage in the team sports (pre-test)/volleyball (post-test) PE lessons …” and includes 24 items (four items per factor) that measure intrinsic regulation (e.g., “because I enjoy learning new skills”; αpre/post = 0.68/0.76), integrated regulation (e.g., “because I believe that physical education is in agreement with my values”; αpre/post = 0.24/0.80), identified regulation (e.g., “because I can learn skills that could be used in other areas of my life”; αpre/post = 0.80/0.82), introjected regulation (e.g., “I feel guilty when I don’t exercise”; αpre/post = 0.70/0.69), external regulation (e.g., “because other people say I should”; αpre/post = 0.73/0.16), and amotivation (e.g., “I don’t see why I should have to exercise”; αpre/post = 0.68/0.77. The items were rated on a 7-point Likert scale, ranging from 1 (“strongly disagree”) to 7 (“strongly agree”). Due to the reduced number of items for each factor, Cronbach’s alpha values above 0.60 can also be accepted [45]. However, integrated and external regulations were removed because Cronbach’s alpha values were lower than 0.60 in the pre-test and post-test, respectively.

##### Intention to be Physically Active

Students’ perceptions of intention to be physically active were assessed using the Spanish version [49] of the theory of planned behavior questionnaire. The scale begins with the question stem “I engage in team sports (pre-test)/volleyball (post-test) PE lessons …” and it is comprised of three items (e.g., “I will participate in volleyball during my free time in the next five weeks”; αpre/post = 0.94/0.89). The scale is rated on a 7-point Likert scale ranging from 1 (“strongly agree”) to 7 (“strongly disagree”).

#### 2.2.2. Qualitative Measurement

Two focus groups of eight students each (e.g., four boys and four girls) were conducted by an external researcher both in the pre-test and the post-test to collect the qualitative data. The 16 students who participated in the first two focus groups in the pre-test remained in the second two focus groups in the post-test. Focus groups lasted for approximately 50 min and were tape-recorded for later transcription with the use of pseudonyms to ensure participant confidentiality. The structure of the interviews in the focus groups was designed by the research group of this study according to the study variables related to SDT. Examples of these questions are provided in Table 1.

### 2.3. Procedure

The research was authorized by an ethics committee (details are omitted for masked review). Prior to starting the research, the school board and the school PE teacher were informed about the nature of the study and the requirements for participation. Subsequently, written informed consent was required from both students and parents, who were also fully notified about the aim and voluntary nature of the study.

The hybrid TGfU/SE volleyball teaching unit was taught by the same male PE teacher in both groups, who had no previous knowledge or experience in the use of TGfU/SE models. Consequently, before starting the volleyball teaching unit, the PE teacher attended 14 h of a training program over four weeks on both pedagogical models. Several experts in pedagogical models and SDT framework designed and implemented the training program (for a summary, see Table 2). During the first week, the PE teacher spent approximately four hours reading manuscripts about TGfU (e.g., [8]), SE (e.g., [50]), and the hybridization of both pedagogical models (e.g., [35]). In the second week, two 2-hmeetings were held with the PE teacher who conducted the intervention in order to answer doubts and clarify information about both models. The PE teacher was given examples of hybrid TGfU/SE teaching units. In the third week, the PE teacher designed the hybrid TGfU/SE volleyball teaching unit with the help of four experts in both pedagogical models, who supervised the structure and learning tasks for four hours. Curricular elements of the teaching unit (e.g., aim, content, task, competences, evaluation, etc.) were established in this phase according to the Spanish PE education curriculum. Finally, in the fourth week, the PE teacher carried out two PE sessions with two different groups of students who did not participate in the current study. These PE sessions were observed and supervised by the four experts in both pedagogical models. After each session, these experts provided extensive feedback that focused not only on the strengths of the sessions but also on the elements of the TGfU/SE models that the PE teacher could improve.

### 2.4. Intervention Program

The TGfU/SE volleyball teaching unit took place twice a week over a period of five weeks (ten 50-min lessons). The structure and the lesson content for this hybrid TGfU/SE volleyball teaching unit are summarized in Table 3. The structure of this teaching unit was designed according to the characteristics of the SE model (e.g., seasons, affiliation, formal competition, record-keeping, final event, and festivity). The teaching unit had three phases: (1) a learning phase (lessons 1–5), (2) a formal competition phase (lessons 6–9), and (3) a final event (lesson 10). In the first lesson of the learning phase, the PE teacher organized persistent mixed-gender teams with different ability levels. Students developed their team identity (e.g., name, image, song, color, and mascot), and their roles in the teaching unit (e.g., coaches, journalists, statisticians, fitness leaders, and captains) in each of the groups.

It is noteworthy that the tasks set by the PE teacher in the learning phase of the SE season were designed according to the features of the TGfU model. In this regard, the teaching-learning process was developed in a contextualized way, based on modified games that maintained the nature of volleyball, as noted in the original aims of SE [10]. Consequently, representative, purposeful, and authentic tasks were presented to the students. Further, learning tasks were adapted to students’ needs, cognitive characteristics, and ability levels using the pedagogical principles of modification-representation and tactical complexity. For instance, cooperative tasks (e.g., 1 + 1 or 2 + 2) were implemented at the beginning of the unit. Subsequently, the tactical and technical complexity of the tasks progressively increased by using a larger number of teammates and opponents (e.g., 1 vs. 1; 1 vs. 1 + 1; 2 vs. 2; 3 vs. 3), and a variety of technical actions, respectively (e.g., set, dig, serve, and spike). The pedagogical principle of modification-exaggeration was also implemented by the PE teacher to modify the volleyball rules, and to emphasize specific tactical learning aims (e.g., to let the ball touch the ground before hitting it).

During this learning phase, the teacher also provided the students with positive feedback about their individual progress and skill development. In addition, the teacher also used open-ended questioning to guide the students to the correct answers to the tactical problem (e.g., Where are the opposing team’s players placed? Where are the free spaces in the opposite court?). These questions were not only used to learn how to solve different tactical problems, but also to facilitate discussion among group members.

When the learning phase was completed, all teams participated in different volleyball matches (3 vs. 3) during the competition phase. The teams and roles adopted by the students in the previous phase were maintained in this second phase. For example, the information provided by the statisticians guided the tournament matches and the classification. In addition, the PE teacher gathered data on team organization, team festivity, team originality, and fair play, and these were embedded into the points the teams accumulated during the competition phase matches.

After the formal competition phase, a final culminating event was carried out to decide the winning teams, which was followed by an awards ceremony. Based on the data obtained by the PE teacher cited above, the following prizes were awarded: winning teams, most original team, most festive team, most organized team, a fair play award, and an award for the most valuable player.

### 2.5. Instructional and Treatment Validity

Following the guidelines of Hastie and Casey [51], the fidelity of the hybrid TGfU/SE teaching unit lessons was assessed using a checklist (see Table 4). Items 1, 2, 4, and 6 permit assessing the PE teacher’s fidelity to the SE model, while items 3, 5, and 7 permit examining the PE teacher’s fidelity to the TGfU model. The fidelity assessment was based on direct and external systematic observation. Following the recommendations of Tabachnick and Fidell [52], two researchers with experience in pedagogical models in PE randomly observed five of the ten volleyball lessons. One hundred percent agreement was reached between the two observers. Each observer confirmed that all key aspects included in the instructional checklist were made by the teachers in each of the observed lessons using the hybrid TGfU/SE unit.

### 2.6. Data Analysis

#### 2.6.1. Quantitative Analysis

Analyses were performed using IBM SPSS Statistics v.24.0 software (SPSS Inc., Chicago, IL, USA). Normality and multicollinearity were tested before conducting the main analyses. Results of the Shapiro–Wilk test revealed that data were normally distributed (*p* > 0.05) and, therefore parametric statistics were used in all analyses. Given that Spearman values were over 0.70 in the study variables in both the pre-test and post-test, the assumption of multicollinearity was deemed to have been met [52].

Descriptive statistics (e.g., mean and standard deviation) were calculated for all study variables for each gender at each of the two different time points (e.g., pre-test and post-test). In addition, Cronbach’s alpha coefficient was conducted to assess reliability of the scales. A 2 × 2 within-test time (e.g., pre-test and post-test) x gender (e.g., boys and girls) MANOVA was conducted. A Bonferroni correction factor was used to control for Type 1 errors in multivariate comparisons. If an overall multivariate effect was significant, the univariate ANOVAs were interpreted for both genders to examine which specific constructs contributed to the overall multivariate effect with Bonferroni corrections applied. Cohen’s criteria were used as indicators of small (0.01), medium (0.06), and large (0.14) effect sizes [53]. The level of statistical significance was established at *p* ≤ 0.05 (95% confidence interval).

#### 2.6.2. Qualitative Analysis

The focus groups transcripts were analyzed by two researchers using the framework method [54] by means of deductive content analysis (e.g., using SDT as a theoretical framework). Based on this, the SDT-related variables (e.g., need-supportive behaviors from PE teacher, BPNs satisfaction, novelty and variety satisfaction, motivation, and intention to be physically active) were defined by the research group of this study [55]. Subsequently, texts within the transcripts, which related to these SDT-related variables, were identified. Categories identified in the initial transcription formed the basis of analysis for the remaining transcripts. The qualitative data were interpreted and organized by the research group into the six SDT-related variables mentioned above. The MAXQDA Analytics Pro software program (VERBI Software, Berlin, Germany) was used to code transcriptions that emerged from the data. To confirm the trustworthiness of the qualitative data, strategies of credibility, transferability, dependability, and confirmability were used [56].

## 3. Results

### 3.1. Quantitative Results

Results indicated a significant main effect of the hybrid TGfU/SE teaching unit with a large effect size (Wilks’ Lambda = 0.101; *F*(15,34) = 20.178; *p* < 0.001; *ηp*^2^ = 0.89). As observed in Table 5, students showed a significant increase in their perceptions of support of the three BPNs from the PE teacher, the satisfaction of the three BPNs, novelty and variety satisfaction, and intrinsic regulation compared to their baseline values. It should be noted that, in the post-test, all study variables showed large effect sizes. In contrast, a significant decrease in intention to participate in volleyball was reported once the volleyball teaching unit was implemented.

A large effect size was found in both boys and girls. Within-group multivariate analysis showed that the effect size was larger in girls (Wilks’ Lambda = 0.154, *F*(15, 34) = 12.411, *p* < 0.001, *ηp*^2^ = 0.84) than in boys (Wilks’ Lambda = 0.211; *F*(15, 34) = 8.463, *p* < 0.001, *ηp*^2^ = 0.78). As can be seen in Table 5, both boys and girls reported significant increases in their perceptions of support of the three BPNs from the PE teacher, the satisfaction of the three BPNs, and novelty and variety satisfaction, compared to their baseline values. In addition, boys reported a significant increase in introjected regulation whereas girls showed a significant increase in intrinsic motivation. Finally, only boys reported a significant decrease in intention to participate in volleyball after the intervention.

### 3.2. Qualitative Results

As observed in Table 6, students who participated in the focus groups at the end of the volleyball teaching unit also supported the results obtained through questionnaires. In the focus groups, students also mentioned improvements in need-supportive teaching behaviors from their PE teacher, with the exception of relatedness support, which remained unchanged. In addition, they also verbalized positive changes in the satisfaction of the three BPNs, novelty and variety satisfaction, and self-determination motivation. Finally, with regard to intention to be physically active, some students expressed their desire to participate in an amateur volleyball team, while others did not.

## 4. Discussion

Grounded in SDT, this pre-experimental study aimed to analyze the effects of a hybrid TGfU/SE volleyball teaching unit on students’ motivational outcomes using a mixed-method approach. Whether the intervention was equally effective for both boys and girls was also analyzed. In this regard, and despite social and cultural stereotypes in team sports in favor of boys [41], it was hypothesized that teaching volleyball in PE through hybrid TGfU/SE unit allows to design a more equitable and inclusive learning environments, where boys and girls would report higher post-test scores on motivational-related variables compared to baseline values. Overall, quantitative and qualitative results showed that after implementing the hybrid TGfU/SE volleyball teaching unit, both boys and girls reported improvements in most of the SDT-related variables. These findings are widely aligned with previous research in PE, based on student-centered pedagogical approaches (e.g., SE and TGfU among others) and hybrid models (e.g., TGfU/SE models), which also reported improvements in students’ motivational outcomes [13,34,36].

In terms of need-supportive behaviors from the PE teacher, self-reported surveys showed that the boys and girls who participated in this study reported higher levels of autonomy, competence, and relatedness support after the hybrid TGfU/SE volleyball teaching unit, compared to baseline values. Students also recognized improvements in need-supportive strategies from their PE teacher, with the exception of relatedness support. Improvements in students’ perceptions of autonomy support from their PE teacher could be explained by the inherent characteristics of both TGfU and SE models. For example, in line with TGfU model, students were encouraged in the learning phase to reflect and to discuss tactical problems with their team. Additionally, in the formal competition phase, the different teams had to agree, discuss, and evaluate their own rules, roles, and tactical strategy. Likewise, and in line with some elements of the SE model, at the beginning of the volleyball teaching unit, students of both genders cooperated with each other to create their own team identity and choose a role that matched their own interests the best (e.g., coaches, journalist, statistician, fitness leader, and captain). Overall, students appeared comfortable in dealing with the level of autonomy encouraged. These findings were also supported by qualitative data, which showed outstanding results for autonomy support (e.g., “We liked being able to choose according to our tastes”). These results demonstrate that the provision of autonomy support in the hybrid TGfU/SE teaching unit may be attributed to opportunities provided to the students to become more involved in their educational experience, such as peer leadership within teams [14,32,34].

With regard to competence support, the PE teacher can design authentic learning tasks based on TGfU pedagogical principles (e.g., representation, exaggeration, and tactical complexity) to adapt to students’ needs and levels of competence [10]. Likewise, when the PE teacher uses the SE model, this gives students the opportunity to carry out the roles that best suit their interests and personal strengths, thus facilitating students’ perceived success [12]. These findings were also reinforced by qualitative data obtained through the focus groups. In these groups, students reported that the tasks or tactical problems were gradual, with the tactical tasks being easier at the beginning of the teaching unit, and then becoming more complex. These findings indicate that this hybrid TGfU/SE model may offer an equitable learning environment where students, regardless of their gender, have the opportunity to demonstrate their skills and effectiveness in completing lesson assignments [32].

Moreover, there were also significantly higher relatedness support scores from all participants when the intervention was completed. This result is not surprising given that in TGfU, the PE teacher implemented strategies such as questioning to stimulate within-team exchanges and the discussion of ideas among group members to solve tactical problems in collaboration with peers, which can potentially increase students’ sense of unity and interaction between students of a different gender [57]. In this learning environment, students worked in small groups and the PE teacher stepped back to specifically observe the group discussions, providing them with positive feedback, and prompting them with more questions [58,59]. In addition, in the SE model, students are organized into mixed-gender teams that are persistent for the entire season, which results in positive feelings of affiliation and social connection with other group members [60,61]. Supporting these findings, and viewing the qualitative data, the students expressed that there was always a good learning environment in the volleyball lessons. These findings highlight that throughout the lessons using the hybrid TGfU/SE unit, students increased their sense of affiliation, because they could identify this type of teacher behavior as an effective strategy to interact with other participants and be accepted socially by other students [62].

On the basis of the theoretical tenets of SDT, when a PE teacher creates a need-supportive learning environment, students show greater needs satisfaction, and in turn greater intrinsic motivation [16,17,18,19,20]. According to these tenets, and in line with past studies in PE [63], the participants—boys and girls—in the present study displayed not only higher levels of need-support after completing a hybrid TGfU/SE unit, but also higher levels of satisfaction of the three BPNs. Qualitative data also reported greater perception of choice by students (e.g., “We liked choosing things because this motivates us”), efficacy (e.g., “We started at the lowest level but we have improved”), and integration (e.g., “I had teammates in the group with a lower level but I helped them”). In line with our results, previous intervention studies in PE have suggested that adopting student-centered pedagogical approaches (e.g., TGfU, SE and hybrid TGfU/SE models) permits designing a need-supportive learning environment and, therefore, improving students’ motivational outcomes [32,33,34,35].

Both boy and girl students also reported higher values in novelty satisfaction after the intervention. The lack of previous experience of students in both hybrid TGfU/SE models and in volleyball in PE lessons could explain these results. For example, consistent with the characteristics of SE model, there were novel situations in PE lessons, such as different roles (e.g., coaches, journalists, statisticians, fitness leaders, and captains), persistent teams with their own identity (e.g., name, image, song, color, and mascot), and a sport season that could explain the results found. Moreover, this volleyball teaching unit was based on small-sided and/or modified games following the pedagogical principles of TGfU model (e.g., representation, exaggeration, and tactical complexity). Therefore, there were a lot of novel exercises during this teaching unit. These possible explanations were supported by data collected through the focus groups (e.g., “I had never been a referee, but I liked this role to learn more about the sport”; “belonging to a team or participating in matches is something we had never done before”). These results are noteworthy because previous experimental studies in PE have shown that experiencing novelty can contribute to higher levels of autonomous motivation among students [64,65,66,67]. From this perspective, the implementation of a learning unit by using a hybrid TGfU/SE model can be essential to create adaptive learning environments that allows students to experience new experiences and sensations that have not been previously experienced. This, in turn, enables them to reach multiple positive cognitive, affective, and behavioral outcomes in PE [24,37,64].

In terms of variety, both boys and girls also displayed higher variety satisfaction after implementing the hybrid TGfU/SE volleyball teaching unit in comparison with their baseline values. These results could be explained by the wide variety of activities that were developed in this hybrid TGfU/SE volleyball teaching unit. For example, consistent with the characteristics of SE model, there were a variety of tasks in the different phases of the season (e.g., learning phase, competition phase, and a final event). Moreover, in line with TGfU model, each session of this volleyball teaching unit was based on a wide variety of small-sided and/or modified games. This is the first study that shows how a hybrid TGfU/SE teaching unit may be useful to improve students’ perception of variety in PE lessons. In this sense, these results should be highlighted because variety has been associated with positive behaviors related to physical exercise [47], and it could also trigger adaptive consequences in the PE context. However, more research is required to obtain stronger evidence of the effects of variety on PE

Further, the hybrid TGfU/SE volleyball teaching unit was also effective in improving the levels of intrinsic motivation among students. These results were supported by quantitative and qualitative data with sentences such as the following: “We like this learning format because it motivates us a lot” or “I wanted to participate because I liked it so much and wanted to improve”. According to SDT, these results could be due to the implementation of the hybrid TGfU/SE model being framed within a need-supportive environment, facilitating individual student development, pleasure, and growth [20,21]. In the gender analysis, these improvements in intrinsic motivation were also significant in girls, and almost significant in boys. In addition, compared to baseline values, boys also displayed higher levels of introjected regulation after finishing the volleyball teaching unit. Engaging in some tasks with the aim of not impairing the performance of their own team or the score given by their peers in the hybrid TGfU/SE teaching unit could explain this increase in introjected regulation.

While SDT tenets ensure that increasing intrinsic motivation should represent an increase in individuals’ adaptive outcomes [21], the results of this study showed that intention to be physically active decreased in boys after implementing the hybrid TGfU/SE volleyball teaching unit. These findings are not in line with a previous study in which students reported a desire to continue participating in sport activities outside school after the hybrid TGfU/SE volleyball teaching unit [35]. Given that volleyball is not a popular sport among Spanish adolescents, and participants had no previous experience in volleyball in PE lessons, we decided to ask students about their experiences in team sports before implementing the hybrid TGfU/SE unit. In this sense, students who participate in popular sports, such as football or basketball, could have responded positively to intention to be physically active in team sports. Yet, when the teaching unit finished, we asked students about their specific intention to play volleyball in their leisure time. Because the majority of the students already practiced other sports and the opportunities to play volleyball in the nearby context were sparse, the students responded negatively to intention to continue practicing this sport outside school. In addition, qualitative data were not conclusive. While some girls said they would like to continue playing volleyball to meet people, most students preferred to engage in other activities. In this sense, research seems necessary to shed more light on the potential effects that different hybrid TGfU/SE teaching units, in distinct contexts, may have on intention to be more active outside the school setting.

The present study has several strengths that distinguish it from previous studies. First, to the best of our knowledge, this is the first study that examines the effects of a hybrid TGfU/SE unit via a mixed-methods approach. This design, which combines quantitative and qualitative approaches, permitted obtaining greater and more in-depth information of the effects of this hybrid TGfU/SE unit on students’ motivational outcomes. Second, the PE teacher’s fidelity in the implementation of the hybrid TGfU/SE teaching unit was assessed. Third, unlike previous studies, this hybrid TGfU/SE volleyball teaching unit was taught by a novel PE teacher who had no previous knowledge or experience in the use of TGfU/SE models. Finally, this is the first study that has examined the effects of this TGfU/SE teaching unit on students’ perception of need-supportive behaviors from PE teachers, as well as novelty and variety satisfaction.

### Limitations and Future Research Directions

While the findings of the current study increase existing research of the positive effects of hybrid TGfU/SE teaching units, there were several limitations that should be considered when interpreting our findings. First, given the absence of a control group, and convenience and small sample size, results should be interpreted with caution. Future studies with representative and larger sample sizes at different schools and with more PE teachers could help to refute these findings. In addition, this would also permit examining the extent to which increases in students’ motivational outcomes are positively related to increases in students’ perception of need-supportive behaviors from PE teachers. Second, the present study only examined the effects of a hybrid TGfU/SE teaching unit on variables related to the “bright side” of motivation (e.g., need-supportive teaching behaviors, need satisfaction, self-determined motivation, and positive outcomes). Examining variables related to the “dark side” of motivation (e.g., need-thwarting behaviors, needs frustration, and negative outcomes) could be a new and interesting avenue of research. Third, the intervention was developed in a team sport such as volleyball that is not very common in PE curricular programs in Spain. Therefore, in the pre-test, students were asked about their perceptions in SDT-related variables in team sports in general, practiced previously in PE, while the post-test would refer solely to volleyball. Fourth, despite intention to be physically active being closely related to physical activity [68], this study did not assess this health-related behavior. Fifth, only one hybrid TGfU/SE teaching unit was developed in the current study. Consequently, it would be valuable to implement other consecutive hybrid TGfU/SE teaching units with different characteristics (e.g., individual sports, outdoor and adventurous activities, artistic expression activities, etc.) and gender stereotypes (e.g., football, dance, skipping rope, rugby, etc.). Finally, it should be noted that the duration of this TGfU/SE teaching unit was in line with the Spanish PE education curriculum, where PE teaching units usually comprise between 8 and 10 lessons. Future studies could increase the length of these hybrid TGfU/SE units to 15–20 lessons to see whether the effect size on motivational outcomes increases after the intervention. Moreover, a follow-up post-intervention should be conducted to examine long-term intervention effects.

## 5. Conclusions

As the PE curriculum in Spain faces further time constraints, it is important for PE teachers to employ the most effective pedagogical approach, which allow to improve students’ motivational responses in PE. In this line, this study’s findings provide initial evidence that adopting an MBP can be a decisive and effective strategy to empower need-supportive behavior from PE. In this regard, using a combination of two pedagogical models such as TGfU/SE helps both boys and girls to enhance the satisfaction of the three basic psychological needs, variety, and novelty, which, in turn, could help to improve students’ motivation in PE lessons. Moreover, and despite the existence of social and cultural stereotypes in team sport such as volleyball in favor of boys, the hybrid TGfU/SE unit was more effective for girls. The characteristics of the SE model and the pedagogical principles of the TGfU model can result in more positive motivational experiences in PE for girls.

## Figures and Tables

**Table 1:** Self-determination theory (SDT) questions asked to the focus groups.

Variable	Factor	Examples of Questions
Basic psychological needs (BPNs) support from PE teacher	Autonomy support	In team sports (pre-test)/volleyball (post-test) lessons, does your PE teacher: - Ask you about your interests and preferences? - Allow you to choose between different exercises or some aspects of the lessons?
	Competence support	In team sports (pre-test)/volleyball (post-test) lessons, does your PE teacher: - Adapt activities to students’ ability levels? - Provide you with positive feedback and questioning? - Encourage you to carry out the exercises?
	Relatedness support	In team sports (pre-test)/volleyball (post-test) lessons, does your PE teacher: - Help to have a good atmosphere in class? - Help you to solve problems within the class?
BPNs, novelty, and variety satisfaction	Autonomy satisfaction	In team sports (pre-test)/volleyball (post-test) lessons: - Do you feel you have opportunities to make choices? - Do you feel you participate in the decision-making process?
	Competence satisfaction	In team sports (pre-test)/volleyball (post-test) lessons: - Do you feel you have improved new skills? - Do you feel you are doing the tasks correctly?
	Relatedness satisfaction	In team sports (pre-test)/volleyball (post-test) lessons: - Do you have positive relationships with your peers? - Do you feel integrated in your peer group?
	Novelty satisfaction	In team sports (pre-test)/volleyball (post-test) lessons: - Do you feel that you do novel activities/lessons or are they all familiar? - Has this volleyball teaching unit been new to you? (post-test)
	Variety satisfaction	In team sports (pre-test)/volleyball (post-test) lessons: - Do you feel that you do varied activities/lessons or are they all similar? - Have the volleyball sessions been varied for you? (post-test)
Motivation	Motivational regulations	In team sports (pre-test)/volleyball (post-test) lessons: - Do you feel motivated to participate in the PE lessons? - What are your main reasons for participating in PE lessons?
Intention to be physically active	Intention to be physically active	- Would you like to continue participating in these sports or in volleyball in the recess or in your free time? - Would you like to join an amateur team? - Would you like to play a game in these sports or in volleyball from time to time?

**Table 2:** Training program for the physical education (PE) teacher.

Week	Content
First week	The PE teacher read manuscripts about teaching games for understanding (TGfU), sport education (SE), and the hybridization of both pedagogical models.
Second week	Two 2-h meetings were conducted with the PE teacher to answer doubts and clarify information about both models.
Third week	The PE teacher and four experts designed the hybrid volleyball teaching unit.
Fourth week	The PE teacher conducted two PE sessions, which were observed and supervised by four experts.

**Table 3:** Season plan for the hybrid teaching games for understanding/sport education (TGfU/SE) volleyball teaching unit.

Lesson	TGfU Component	SE Component
1	Teacher-directed instruction: 1 + 1 overhand pass (cooperative).	Introduction to the concept of the season. Explanation of the model and competition format. Assignment of teams and roles. Development of team identity. Teacher-directed instruction within-team practice.
2	1 + 1 overhand pass (cooperative). 1 vs. 1 overhand pass.	Teacher-directed instruction within-team practice. Introduction to team roles and responsibilities (photographer, captain, coach, fitness leader, and statistician). For example, fitness leaders conduct warm-up and cool-down, and coaches and captains design some learning tasks.
3	1 vs. 1 + 1 overhand pass. 2 + 2 overhand pass (cooperative).	
4	2 vs. 2—Serve and overhand pass. 2 vs. 2 + 1—Serve and overhand pass with questioning.	
5	3 vs. 3—Serve and overhand pass. 3 vs. 3—Serve, overhand pass, and forearm touch with questioning (e.g., Where are the opposing team’s players placed?)	
6	3 vs. 3—Serve, overhand pass, and forearm touch with questioning (e.g., Where are the free spaces in the opposite court?).	Championships for season points. Student-directed instruction. Scrimmages with the opposing teams. Duty team responsibilities (photographer, captain, coach, fitness leader, and statistician). For example, fitness leader conducted the warm-up and cool-down. The statisticians collected data about some aspects of the game (e.g., number of games won, number of points earned per player, failure to comply with the rules, etc.). Photographers took pictures to be published on school’s website.
7		
8		
9		
10	Culminating event and awards.	Culminating event and festivity.

**Table 4:** Instructional check list.

Date:	Present	Absent
1. Students go to a designated home area and begin to warm up with their own group/team.		
2. Students keep performance records.		
3. All the lessons’ tasks are related to the small-sided game that is being taught.		
4. Students perform specialized roles within their group/team.		
5. Modifications to the full game are performed.		
6. Students’ individual performance scores count for a formal and public scoring system.		
7. Students dedicated at least 30 min of the lesson to playing modified games.		

**Table 5:** Descriptive statistics and intervention effects on motivational outcomes.

Variables	Gender	Pre-Test (I)		Post-Test (J)		Mean Difference (J–I)	Standard Error	*F*	*p*	*η* *p* ^2^	95% CID	
		*M*	*SD*	*M*	*SD*						LL	UL
Autonomy support from PE teacher	Total	2.73	0.65	3.89	0.52	1.16	102.31	102.31	<0.001	0.676	0.93	1.39
	Boys	2.74	0.56	3.78	0.55	1.04	41.20	41.20	<0.001	0.462	0.71	1.36
	Girls	2.73	0.76	4.01	0.49	1.28	62.41	62.41	<0.001	0.565	0.95	1.60
Competence support from PE teacher	Total	3.34	0.72	4.27	0.47	0.93	21.39	21.39	<0.001	0.587	0.70	1.14
	Boys	3.43	0.74	4.16	0.51	0.73	13.52	13.52	0.001	0.220	0.42	1.03
	Girls	3.26	0.65	4.38	0.41	1.12	36.35	36.35	<0.001	0.431	0.81	1.42
Relatedness support from PE teacher	Total	3.67	0.70	4.32	0.54	0.65	10.40	10.40	<0.001	0.337	0.38	0.90
	Boys	3.65	0.81	4.25	0.58	0.60	10.59	10.59	0.002	0.181	0.22	0.97
	Girls	3.70	0.62	4.39	0.50	0.69	14.00	14.00	<0.001	0.226	0.31	1.06
Autonomy satisfaction	Total	2.62	0.66	3.87	0.50	1.25	144.45	144.45	<0.001	0.747	1.04	1.45
	Boys	2.79	0.62	3.85	0.50	1.06	54.59	54.59	<0.001	0.532	0.77	1.34
	Girls	2.45	0.69	3.89	0.48	1.44	100.76	100.76	<0.001	0.677	1.15	1.72
Competence satisfaction	Total	3.46	0.93	4.09	0.56	0.63	34.60	34.60	<0.001	0.414	0.41	0.83
	Boys	3.55	0.86	4.09	0.68	0.54	13.52	13.52	0.001	0.220	0.23	0.84
	Girls	3.38	0.73	4.09	0.62	0.71	36.35	36.35	<0.001	0.431	0.40	1.01
Relatedness satisfaction	Total	3.77	0.78	4.33	0.64	0.56	16.08	16.08	<0.001	0.247	0.27	0.83
	Boys	3.84	0.93	4.30	0.53	0.46	3.95	3.95	0.024	0.076	0.06	0.85
	Girls	3.71	0.97	4.36	0.59	0.65	10.86	10.86	0.002	0.185	0.25	1.04
Novelty satisfaction	Total	2.75	0.65	4.09	0.52	1.34	196.49	196.49	<0.001	0.800	1.14	1.52
	Boys	2.92	0.54	4.08	0.48	1.16	78.14	78.14	<0.001	0.619	0.89	1.42
	Girls	2.59	0.70	4.10	0.57	1.51	131.82	131.82	<0.001	0.733	1.24	1.77
Variety satisfaction	Total	3.02	0.64	4.08	0.47	1.06	106.04	106.04	<0.001	0.684	0.85	1.25
	Boys	3.18	0.56	4.05	0.42	0.87	37.90	37.90	<0.001	0.441	0.58	1.15
	Girls	2.87	0.70	4.11	0.52	1.24	76.65	76.65	<0.001	0.615	0.95	1.52
Intrinsic regulation	Total	4.46	0.89	5.01	1.01	0.55	12.44	12.44	<0.001	0.203	0.23	0.85
	Boys	4.43	0.90	4.86	0.96	0.53	3.83	3.83	0.056	0.074	−0.01	0.87
	Girls	4.50	0.90	5.16	1.07	0.66	9.04	9.04	0.004	0.159	0.21	1.10
Identified regulation	Total	4.18	2.05	4.27	1.12	0.09	0.05	0.05	0.816	0.001	−0.69	0.54
	Boys	4.90	2.87	4.63	1.28	−0.27	0.38	0.38	0.539	0.008	−1.14	0.60
	Girls	4.76	0.89	4.89	0.95	0.13	0.08	0.08	0.773	0.002	−0.75	1.01
Introjected regulation	Total	3.93	1.21	4.35	1.13	0.42	3.44	3.44	0.069	0.066	−0.02	0.85
	Boys	3.72	1.24	4.46	1.42	0.74	5.60	5.60	0.022	0.105	0.11	1.36
	Girls	4.15	1.10	4.24	0.78	0.09	0.08	0.08	0.775	0.002	−0.53	0.71
Amotivation	Total	3.43	1.15	3.81	1.28	0.38	2.69	2.69	0.107	0.052	−0.08	0.85
	Boys	3.38	1.08	3.92	1.39	0.54	2.63	2.63	0.112	0.052	−0.13	1.21
	Girls	3.48	1.22	3.71	1.21	0.23	0.47	0.47	0.494	0.010	−0.44	0.90
Intention to be physically active	Total	4.13	1.71	2.82	1.39	−1.31	16.19	16.19	<0.001	0.248	−1.96	−0.66
	Boys	4.42	1.86	2.68	1.18	−1.74	14.55	14.55	<0.001	0.233	−0.26	−0.82
	Girls	3.84	1.61	2.96	1.57	−0.88	3.69	3.69	0.061	0.071	−1.80	0.04

*Note:* M = mean; SD = standard deviation; CID = confidence interval differences; LL = lower limit; UL = upper limit; level of statistical significance was established at *p* ≤ 0.05.

**Table 6:** Students’ qualitative responses about study variables before and after conducting the TGfU/SE hybrid unit.

Variable	Descriptor	Factor	Sample Quote in the Pre-Test	Sample Quote in the Post-Test
BPN support from PE teacher	Students perceived a positive change in autonomy and competence support of the PE teacher. However, students did not report an improvement in relatedness support.	Autonomy support (+)	“They did not let us choose anything at all” (Girl 1, FG 1) “They occasionally asked us what sport we wanted to do” (Boy 1, FG 2)	“We liked it when they allowed us to choose different things” (Boy 3, FG 1) “We liked being able to choose according to our tastes” (Boy 5, FG 2)
		Competence support (+)	“The exercises were too easy” (Girl 4, FG 1) “The tasks were achievable, normal” (Boy 3, FG 2)	“The exercise tasks have been gradual, easy to begin with and then more difficult at the end” (Girl 1, FG 1) “The tasks depended on the session we were doing” (Boy 3, FG 2)
		Relatedness support (=)	“It was normally good, but during the matches there were grudges due to the competitiveness, they only wanted to win” (Boy 3, FG 2) “The teacher created a good atmosphere because we didn’t get on well in class” (Girl 1, FG 1)	“The teacher fostered a good atmosphere in class” (Girl 4, FG 1) “There was always a good atmosphere in volleyball classes” (Boy 1, FG 2)
BPN, novelty, and variety satisfaction	Students perceived a positive change in the satisfaction of their three BPNs. In addition, they also reported improvements in novelty and variety satisfaction.	Autonomy satisfaction (+)	“We would have liked to choose a series of exercises to be able to do them” (Boy 3, FG 1) “We would have had a better time playing things we like” (Boy 4, FG 2)	“We liked being able to choose the name, t-shirt color, reaching an agreement among all of us” (Boy 1, FG 2) “We liked being able to choose things because it motivates us” (Girl 4, FG 1)
		Competence satisfaction (+)	“I’m no good because I’m a bad shot, not good at running, handling balls… I’m better at individual sports” (Boy 2, FG 1) “I’m not good at them because I’ve never practiced them” (Boy 1, FG 2)	“We are much better at it than when we began, because we have learnt things” (Boy 3, FG 2) “We started at the lowest level, but we have gradually improved” (Girl 1, FG 1)
		Relatedness satisfaction (+)	“Yes, because they thought they were superior, you’re scared of failing because they blame you” (Boy 4, FG 1) “It depends on who you play with, they could benefit or harm you in your grades” (Boy 3, FG 2)	“We like working with other colleagues who we did not know so well” (Girl 1, FG 1) “I had girls in the group who did not do much, but I tried to help them” (Boy 3, FG 2)
		Novelty satisfaction (+)	“We were already familiar with everything because we’d done it before, in previous years, from primary school; we already did sport, it was always basic” (Boy 1, FG 2) “We always repeated sports that we were already familiar with” (Girl 4, FG 1)	“Organizing teams, roles, tournaments, and matches because we had never done this in sports” (Boy 1, FG 2) “We had never refereed, but we liked it, to learn more about the sport” (Girl 4, FG 1)
		Variety satisfaction (+)	“It was always the same model of classes” (Boy 3, FG 2) “They were very similar because the exercises we repeated” (Girl 4, FG 1)	“There were different types of activities” (Boy 5, FG 1) “The matches were repetitive, but the exercises changed” (Girl 5, FG 2)
Motivation	Students perceived more self-determined forms of motivation to engage in PE lessons.	Motivational regulations (+)	“We used to go to class because if not, they gave us a fail” (Girl 5, FG 2) “We used to go to class because it was compulsory” (Boy 3, FG 1)	“With this format, we liked it and it motivated us more” (Boy 4, FG 2) “I wanted to participate because I liked it and wanted to improve” (Girl 1, FG 1)
Intention to be physically active	Some students indicated that they would not play volleyball at recess, while others expressed their intention to participate in volleyball in a federated team in the future.	Intention to be physically active (+/−)	“No, because it’s not the same doing something for pleasure as for obligation” (Boy 5, FG 1) “I don’t want to play because I prefer spending my time on other things” (Girl 1, FG 1) “We don’t feel like playing during recess, we prefer to eat or talk” (Girl 3, FG 2)”	“To be honest, we would not play during recess” (Girl 2, FG 2) “We would like to play in a team to get to know people” (Girl 5, FG 2) “Depending on the day, yes I would like to play” (Boy 3, FG 1)

*Note*. FG = focus group; the following symbols (+ or −) refer to the positive (+) or negative (−) students’ perceptions. The interventions that were perceived both positively and negatively by the students were highlighted as follows: (+ or −).

## Data Availability

The data presented in this study are available on request from the corresponding author.
